# Bioelectricity versus bioethanol from sugarcane bagasse: is it worth being flexible?

**DOI:** 10.1186/1754-6834-6-142

**Published:** 2013-10-03

**Authors:** Felipe F Furlan, Renato Tonon Filho, Fabio HPB Pinto, Caliane BB Costa, Antonio JG Cruz, Raquel LC Giordano, Roberto C Giordano

**Affiliations:** 1 Chemical Engineering Graduate Program, Federal University of São Carlos, PPGEQ/UFSCar Via Washington Luis, km 235, São Carlos, SP, Brazil; 2 Department of Chemical Engineering, Federal University of São Carlos, DEQ/UFSCar Via Washington Luis, km 235, São Carlos, SP, Brazil

**Keywords:** Second generation ethanol production, Techno-economic evaluation, Lignocellulose, Sugarcane, Bagasse, Process simulation

## Abstract

**Background:**

Sugarcane is the most efficient crop for production of (1G) ethanol. Additionally, sugarcane bagasse can be used to produce (2G) ethanol. However, the manufacture of 2G ethanol in large scale is not a consolidated process yet. Thus, a detailed economic analysis, based on consistent simulations of the process, is worthwhile. Moreover, both ethanol and electric energy markets have been extremely volatile in Brazil, which suggests that a flexible biorefinery, able to switch between 2G ethanol and electric energy production, could be an option to absorb fluctuations in relative prices. Simulations of three cases were run using the software EMSO: production of 1G ethanol + electric energy, of 1G + 2G ethanol and a flexible biorefinery. Bagasse for 2G ethanol was pretreated with a weak acid solution, followed by enzymatic hydrolysis, while 50% of sugarcane trash (mostly leaves) was used as surplus fuel.

**Results:**

With maximum diversion of bagasse to 2G ethanol (74% of the total), an increase of 25.8% in ethanol production (reaching 115.2 L/tonne of sugarcane) was achieved. An increase of 21.1% in the current ethanol price would be enough to make all three biorefineries economically viable (11.5% for the 1G + 2G dedicated biorefinery). For 2012 prices, the flexible biorefinery presented a lower Internal Rate of Return (IRR) than the 1G + 2G dedicated biorefinery. The impact of electric energy prices (auction and spot market) and of enzyme costs on the IRR was not as significant as it would be expected.

**Conclusions:**

For current market prices in Brazil, not even production of 1G bioethanol is economically feasible. However, the 1G + 2G dedicated biorefinery is closer to feasibility than the conventional 1G + electric energy industrial plant. Besides, the IRR of the 1G + 2G biorefinery is more sensitive with respect to the price of ethanol, and an increase of 11.5% in this value would be enough to achieve feasibility. The ability of the flexible biorefinery to take advantage of seasonal fluctuations does not make up for its higher investment cost, in the present scenario.

## Background

It is already a consensus that a shift of the global energy matrix towards renewable sources is mandatory. Yet, the role that each specific alternative will play, say, at the year 2050, will be defined along the road, depending on technological developments, political options by stakeholders, economical and social demands. Anyway, in this scenario ethanol will certainly be an important biofuel.

Sugarcane is known to be the most efficient crop for 1G ethanol production, with an energy balance of 9.3 produced/consumed tonne of oil equivalent (toe) [1]. During the 1970’s the Brazilian government initiated the National Ethanol Program (PROALCOOL, in Portuguese, [2]) to decrease national dependence on oil. Since then, the use of 1G ethanol as a vehicle fuel has been consolidated, and presently 86% of the cars sold in this country are flex-fuel, running with any mixture of ethanol and gasoline [3]. In modern facilities, ethanol production is a highly integrated process, with sugarcane bagasse burnt in boilers to supply the industrial plant energy demands, further exporting the surplus of electric energy to the grid.

One of the alternatives for the industrial production of 2G ethanol is the biochemical route, i.e., acid or enzymatic hydrolysis of the biomass followed by fermentation of the resulting sugars. Logistics and transportation of the lignocellulosic raw material may be a bottleneck for 2G ethanol [4]. From this point of view, sugarcane bagasse has an important advantage, since it has already been collected and processed for the extraction of the juice, being immediately available at the plant site. Moreover, sugarcane trash (mostly the leaves) can be transported with the stalk, after small adaptations of the mechanical harvesting – although part of this biomass must be left for covering the fields [5]. Since the process must be energetically self-sufficient, the addition of sugarcane trash as boiler fuel can increase the amount of bagasse available for hydrolysis, therefore enhancing ethanol yields.

Industrial production of 2G ethanol is still not consolidated in large scale. Therefore, an economic analysis is important to indicate if it is the most interesting alternative, specially when compared to selling electric energy (bioelectricity). Nevertheless, this answer is not unique, given the volatility of relative prices: biomass electricity prices in public auctions in Brazil ranged from 85.35 USD/MWh to 53.02 USD/MWh in the last two auctions (Aug/2010 and Aug/2011) [6], a -37.9% variation. The spot market (that buys surplus power, beyond the amount contracted during the auctions) presented an even higher range of prices, between 3.26 USD/MWh and 341.13 USD/MWh over the last nine years (Jan/2003 - Dez/2012) [7]. Ethanol prices are equally volatile, changing from 0.258 USD/L to 0.818 USD/L (for the hydrated fuel) over the same period of nine years [8]. All prices above, used in this study, were calculated in Brazilian reais, brought to December/2012 value (to take into account the inflation in the period), and converted to US dollars using the exchange rate value of 2.077 BRL/USD (dez/2012).

Sugarcane biorefineries have been intensively studied by the recent literature. Seabra *et al.* (2011) [9] presented economic and environmental analyses of a sugarcane biorefinery. The authors concluded that, although electric energy presented a better economic feasibility, its environmental impact was greater than the one for second generation ethanol. An economic analysis comparing 2G ethanol production with electric energy was also done by Dias *et al.* (2011) [10]. The authors concluded that, although for the present technology electric energy is a better option, 2G ethanol can compete with it if sugarcane trash is used, provided that new technologies could increase yields. On the other hand, Macrelli *et al.* (2012) [11] presented results for several sugarcane biorefineries configurations and concluded that 2G ethanol from sugarcane is already competitive with 1G starch-based ethanol in Europe. The advantages of the integrated production of 1G and 2G ethanol production based on sugarcane was highlighted by Dias *et al.* (2012) [12]. The integrated biorefinery presented higher ethanol production rates, and better economic and environmental performance, when compared to a stand-alone 2G ethanol-from-sugarcane bagasse plant.

The 2G biorefinery could be flexible, just as the industry that employs current 1G technology already is, shifting between sugar and ethanol production. This new flexible biorefinery might be able to choose between electric energy and 2G ethanol production. The present study focuses on assessing the economic feasibility of a flexible biorefinery, for an autonomous distillery (not considering the manufacture of sugar), and comparing it to the dedicated 1G + electric energy and 1G + 2G ethanol biorefineries. The process chosen as case study uses enzymatic hydrolysis of the biomass (sugarcane bagasse) and ethanolic fermentation of hexoses and pentoses. Specifically, this study presents a computational applicative that may be a useful tool for the process scheduling of future cane-based biorefineries but, beyond that scope, that may also support decision-making concerning national energy policies. Such computationally robust tool was developed within an equation-oriented simulator (EMSO) [13] and is based on phenomenological modelling, at least for the most important unit operations and reactors that are present in the process.

## Results and discussion

### Process simulation

**Figure 1:**
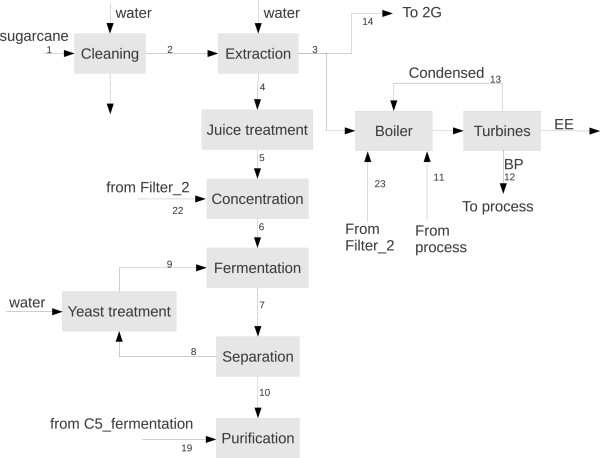
Process diagram for first generation ethanol production.

**Figure 2:**
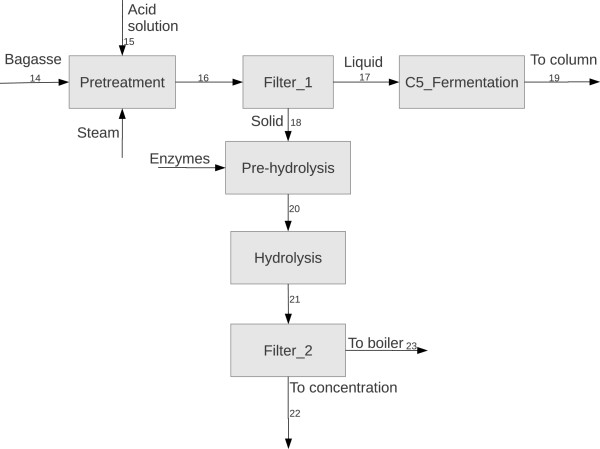
Process diagram for second generation ethanol production.

Two boundary process configurations were considered and compared: an industry producing 1G ethanol and burning all sugarcane bagasse and 50% of the trash produced in the field for power generation in a Rankine cicle (BioEE) and another one using all bagasse surplus (the biorefinery must be energetically self-sufficient) for 2G ethanol production, integrated to the 1G facility (BioEth). The most important information for BioEE and BioEth streams (as enumerated in Figure 1 and Figure 2) is presented in Table 1 and Table 2. 74% of the bagasse could be diverted to 2G ethanol in BioEth. This accounted for an increase in ethanol production of 25.8% when compared to BioEE. At this condition, a specific production yield for 2G ethanol of 120.7 L/tonne of bagasse was obtained, which is a conservative estimate based on current yields (158 L/tonne of lignocellulosic material [12]). The increase in steam consumption for 2G ethanol was entirely fulfilled by the burning of lignin and of non-hydrolyzed cellulose. Since 65% of the cellulose was hydrolyzed, 35% of the material was still able to be separated and used as fuel. Table 3 shows ethanol production (total and specific) for both cases.

**Table 1:** Main streams for 1G + Cogeneration biorefinery (BioEE)

**Stream n°**	**Mass** **flow (kg/h)**	**Temperature (°C)**	**Pressure** **(bar)**	**Fraction of** **sugars**
1	500000	30	1	0.145
2	495675	30	1	0.144
3	131791	30	1	0.02
4	512584	30	1	0.134
5	513565	90	1	0.133
6	389697	111.7	1	0.177
7	578699	31	1	0
8	90016	31	1	0
9	189003	30	1	0
10	488683	31	1	0
11	299182	110.5	2.5	0
12	293196	189.3	2.5	0
13	193026	55.7	0.1	0

**Table 2:** Main streams for 1G + 2G biorefinery (BioEth)

**Stream n°**	**Mass** **flow (kg/h)**	**Temperature (°C)**	**Pressure** **(bar)**	**Fraction of** **sugars**
1	500000	30	1	0.145
2	495675	30	1	0.144
3	131791	30	1	0.02
4	512584	30	1	0.134
5	513565	90	1	0.133
6	332396	111.7	1	0.206
7	493607	31	1	0
8	76920.5	31	1	0
9	161212	30	1	0
10	416687	31	1	0
11	196577	110.5	2.5	0
12	188785	189.3	2.5	0
13	0	55.7	0.1	0
14	97613.3	30	1	0.02
15	174018	30	1	0
16	271631	120	2	0.02
17	201087	100	1	0.02
18	70544	100	1	0.01
19	201045	27	1	0.02
20	153036	50	1	0.02
21	153036	50	1	0.08
22	104925	50	1	0.09
23	48111	50	1	0.05

**Table 3:** First and second generation ethanol production rates

	**BioEE**	**BioEth**
Ethanol production (L/h)	45796.6	57580.8
Specific ethanol production (1G + 2G)	91.6	115.2
(L/tonne of sugarcane (TC))		
2G ethanol production (L/h)	0	11784.2
Specific 2G ethanol production	0	120.7
(L/tonne of bagasse (TB))		

Plant capacity: 500 tonnes of sugarcane per hour.

As shown in Table 4, the steam demand increased by 56.3% from BioEE to BioEth. This represented a steam consumption of 8.8 kg of steam/L of 2G ethanol, compared to 4.0 kg of steam/L of 1G ethanol. This higher consumption was mainly caused by the low concentration of both glucose in the hydrolyzed liquor (9 wt% compared to 17.7 wt% for sugarcane juice) and of ethanol in the C5 wine (21.3 g/L compared to 78.8 g/L for C6 wine). Nevertheless, the higher energy demand of 2G ethanol was diluted by the 1G’s, and the overall specific steam consumption was 5.0 kg of steam/L of ethanol (1G + 2G).

**Table 4:** Steam consumption (total and specific)

**Sector**	**Steam consumption (total (kg/h) / specific (kg/TC))**	
	**BioEE**	**BioEth**
Juice treatment	51971/103.9 ^*a*^	51971/103.9 ^*a*^
Concentration	185240/370.5	241210/482.6
Distillation	121775/243.5 ^*a*^	188455/376.9 ^*b*^
Pretreatment*	0	38493/394.3
**Total**	**185240/370.5**	**289550/579.1**

^*^Steam specific consumption in kg/TB.

^a^Uses steam from concentration step.

^b^Uses both steam from concentration step and from the back pressure turbine (5.2% of the latter).

Plant capacity: 500 tonnes of sugarcane per hour.

Table 5 presents the energy demand by plant sector. Since not all electric power produced was consumed in BioEth, it delivered electric energy to the grid, too. It is clear that the impact of the production of 2G ethanol on the overall energy demand was low. The major impact, in fact, was on the condensing turbine, since all bagasse surplus was diverted to 2G ethanol production. Therefore, this turbine was absent in the BioEth plant.

**Table 5:** Power consumption divided by sector (positive values for produced energy and negative for consumed)

**Sector**	**Power demand/production (kW)**	
	**BioEE**	**BioEth**
Mills	-6368.7	-6368.7
Pretreatment	0	-62.0
Hydrolysis	0	-246.4
Centrifuges	-380.9	-599.6
Pumps	-1389.8	-1055.9
Back pressure turbine	+32106.7	+49863.9
Condensing turbine	+48051.6	0
**Total**	**+72019**	**+41531.3**

### Economic analysis

An economic analysis was performed for both process configurations described above (BioEE and BioEth). Besides, a flexible biorefinery (Flex), which can switch between cogeneration and 2G ethanol, was also considered. This option might enable a better exploitation of the seasonality of both ethanol and electric energy prices (Table 6).

**Table 6:** Ethanol and electric energy average seasonality over the period 2003-2012

**Month**	**Ethanol (%)**	**Electric energy (spot market) (%)**
January	11.19%	22.97%
February	7.64%	-24.71%
March	9.29%	-27.39%
April	5.76%	-31.52%
May	-8.90%	-26.25%
June	-12.61%	-16.08%
July	-8.79%	-5.77%
August	-6.53%	-11.77%
September	-5.37%	20.39%
October	-1.11%	40.86%
November	2.81%	41.92%
December	6.60%	17.36%

Percentage variation from the average mean.

Since the condensing turbine is not necessary for BioEth and, moreover, steam production in this case was lower than in BioEE, the investment in the combined heat and power plant decreased from BioEE to BioEth. On the other hand, an increase in costs for fermentation, distillation and tankage was necessary in BioEth to account for the higher ethanol production. For the flexible biorefinery, it must be suitable for both maximum ethanol and maximum electric energy production, which makes its investment costs the highest. Table 7 presents the investment costs for the cases considered.

**Table 7:** Investment costs by sector of the biorefinery, internal rate of return and net present value

**Sector**	**Cost (10**^**6**^** USD)**		
	**BioEE**	**BioEth**	**Flex**
Sugarcane reception, preparation and milling	38.5	38.5	38.5
Combined heat and power plant	50.2	42.9	50.2
Fermentation, distillation and tankage	30.8	35.4	35.4
Sugarcane juice treatment	23.1	23.1	23.1
Piping, general tankage and valves	15.4	15.4	15.4
Licenses, project and ground leveling	7.7	7.7	7.7
2G (pre-treatment, hydrolysis and C5 fermentation)	0	9.6	9.6
**Total**	**165.8**	**172.7**	**180.0**
IRR^*^	**7.6%**	**8.3%**	**8.0%**
**NPV (10**^**6**^ USD)^*^	**-34.5**	**-30.0**	**-41.8**

^*^for an ethanol price of 513.7 USD/m^3^.

The flexible biorefinery (Flex) allows the decision (considered here to be in a monthly basis) to operate between the two boundary cases represented by BioEE and BioEth. Table 8 shows the chosen option (between electric energy or 2G ethanol) over the whole period considered. It is worth mentioning that both earnings and costs were equally distributed through the year for the flexible biorefinery. Therefore, ethanol and/or bagasse must be stocked to assure the selling during the off-season period. As reported by Agblevor et al. [14], bagasse composition is not greatly affected by storage period, even when it is exposed to weather conditions. The authors verified that only the upper third and the outer region of the dry interior were attacked by micro-organisms and had their composition changed in a period of 26 weeks.

**Table 8:** Chosen option between electric energy surplus (EE) and 2G ethanol production (2G) for the flexible biorefinery

**Month**	**1**^**st**^** to 8**^**th**^** year**	**9**^**th**^** to 13**^**th**^** year**	**14**^**th**^** to 25**^**th**^** year**
January	2G	2G	2G
February	2G	2G	2G
March	2G	2G	2G
April	2G	2G	2G
May	2G	2G	2G
June	2G	2G	2G
July	2G	2G	2G
August	2G	2G	2G
September	EE	2G	2G
October	EE	EE	2G
November	EE	2G	2G
December	2G	2G	2G

The numbers in the first row are the interval of years for which the behavior of the flexible biorefinery remained constant. Spot energy price assumed to be equal to twice its current value (80.2 USD/MWh).

The results of the economic analysis are presented in Table 7. Both IRR and Net Present Value (NPV) showed similar results, with BioEth being the best option, followed by the flexible biorefinery. As it can be seen, none of the options presented a positive NPV or, equivalently, an IRR higher than the Minimum Acceptable Rate of Return (MARR), assumed to be 11%/yr, when the present market prices for ethanol and electric energy in Brazil are considered. Nevertheless, as it will be shown later in the sensitivity analysis, an increase in ethanol price of 21.1% can turn all options feasible. Besides, specifically for the BioEth biorefinery, an increase of only 11.5% in this price would assure feasibility.

Unfortunately, a direct comparison of the obtained result with the ones from literature is not a straightforward task. There is a large variability in the economic premisses and in the technical solutions for the biorefineries that can be considered in a techno-economic study. For example, Seabra and Macedo (2011) [9] considered a 2G biorefinery adjacent to the 1G industrial plant (i.e., not sharing utilities and equipment, just importing bagasse), while Dias et al. (2011) [10] and Macrelli et al. (2012) [11] considered that 1G and 2G production plants were integrated (in different degrees). Even when similar processes are considered, the results can be quite different: while the autonomous 1G industrial plant in Dias et al. (2011) [10] obtained an IRR of 15.9 %, in Macrelli et al. (2012) [11] a similar plant obtained an IRR of 32.1 %.

### Sensitivity analysis

Since ethanol prices presented an approximately normal distribution, its standard deviation was calculated and a variation equivalent to one standard deviation (20.5%) was used for the sensitivity analysis. On the other hand, electric energy price (spot market) did not present a normal distribution and its influence was better seen when the double of its current price was considered. Therefore, for this case a variation of 40% was considered and, for the other ones that had only punctual data available, a variation of 20% was chosen, thus assuming a percent variation similar to the one in ethanol prices. For Flex sensitivity analysis, the variations described were applied before the simulation of the seasonality effect on the prices.

**Figure 3:**
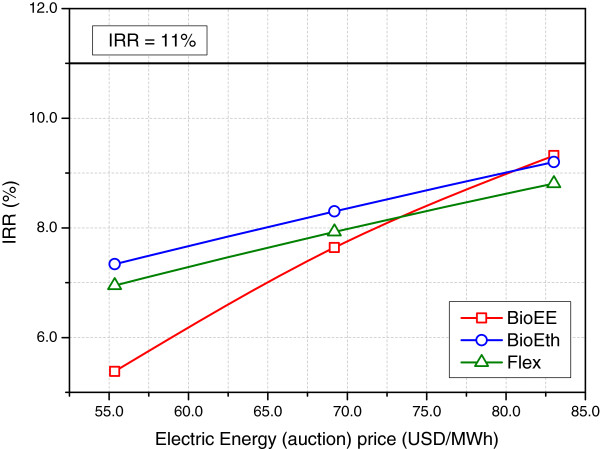
**Impact of electric energy selling prices (annual auctions) on the internal rate of return.** All other prices kept unchanged.

Figure 3 presents the sensitivity analysis of electric energy selling prices in public auctions on the IRR. As expected, the influence was higher for BioEE, since it has the higher amount of electric energy being sold in public auctions. BioEth and Flex produced the same amount of electric energy to be sold in the auction market. Therefore, both were equally influenced by the variation in auction prices. As it can be seen, the effect of the electric energy price on the biorefineries economic performance was not strong enough to make them feasible. The cash flow provided by the selling of electric energy was one order of magnitude smaller than the one for ethanol.

**Figure 4:**
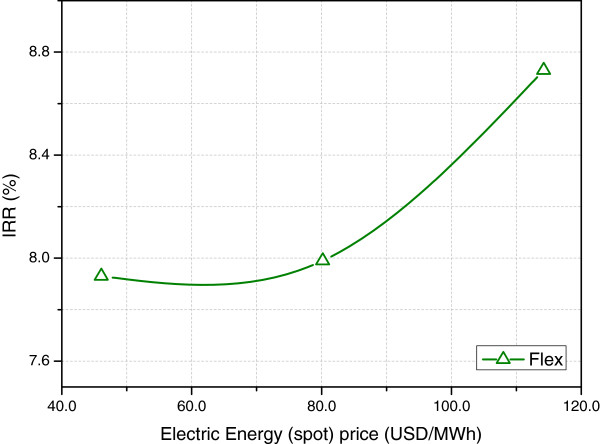
**Impact of electric energy selling prices (spot market) on the internal rate of return.** The central point corresponds to a price (80.2 USD/MWh) equal to twice the current value. All other prices kept unchanged.

Electric energy prices in the spot market only influenced Flex’s IRR (Figure 4). The sensitivity to this parameter was quite small, though. As seen in Table 8, only a few months were dedicated to electric energy production, even when the spot market price was assumed equal to twice its current value. The income from electric energy sold in the spot market was small, compared to the one coming from public auctions. It should be mentioned that an increase in both auction and spot prices can be expected in the near future in Brazil. Thermoelectric plants using natural gas currently complement the production of hydroelectric energy during the dry period, and this is hardly sustainable, both in economic and environmental perspectives. Therefore, an increase in these prices, associated with improvements in the distribution grid, could stimulate investments in cogeneration.

It is worth noticing that while Flex could reproduce almost perfectly the behaviour of BioEth (except for the higher investment cost), the same was not true for BioEE. This is due to the fact that the latter sold all its available electric energy in the auction market, which pays higher prices (in general), while the flexible biorefinery would not do this.

**Figure 5:**
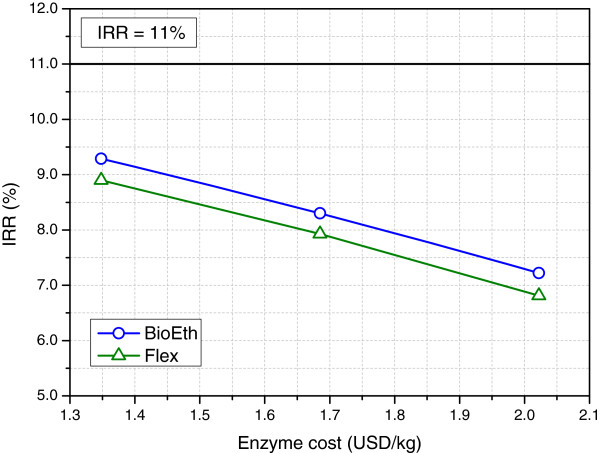
**Impact of enzyme costs on the internal rate of return.** All other prices kept unchanged.

The sensitivity with respect to enzyme prices was not as significant as initially expected (Figure 5). Although enzyme costs played a major role in composing 2G ethanol prices (75% of the total cost), this influence was diluted by the 1G ethanol costs. Therefore, the overall ethanol costs (1G + 2G) changed from 416.3 USD/m^3^ (for an enzyme price of 2.02 USD/kg) to 392.6 USD/m^3^ (for an enzyme price of 1.35 USD/kg), a variation of 5.9%, for a 40% reduction in enzyme price.

**Figure 6:**
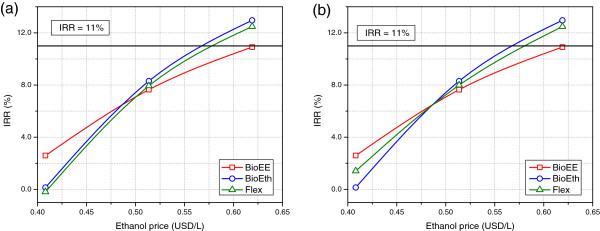
**Impact of ethanol selling prices on the internal rate of return.** In **(a)**, the current spot energy price (40.1 USD/MWh) is considered, while in **(b)** a spot energy price of twice this value (80.2 USD/MWh) is used. All other prices kept unchanged.

Figure 6 shows the impact of ethanol selling prices on the IRR for the current price for electric energy in spot market (a) and for a price equal to twice its current value (b). It is clear that ethanol price presented the higher influence for all biorefineries. In fact, it was the only factor strong enough to make the biorefineries economically feasible, within the range spanned in this study. Accordingly, BioEE, BioEth and Flex became feasible for an ethanol price of 622.1 USD/m^3^, 572.8 USD/m^3^ and 583.1 USD/m^3^, respectively. This means an increase in ethanol price of 21.1%, 11.5% and 13.5%, respectively. While in Figure 6 (a) it is clear that BioEth was always superior to the flexible biorefinery, when a higher price in the spot market was considered (Figure 6 (b)) the flexible biorefinery became less influenced by negative variations of the ethanol price. The switch towards electric energy production for the flexible biorefinery is clear in this case. If the current ethanol price were considered, no extra electric energy was produced in any month for the whole period considered (25 years). On the other hand, when ethanol price is on its lower value, the flexible biorefinery switched to electric energy production during 30% of the months, becoming superior to BioEth.

The non-linear behaviour of the system becomes evident in Figure 6. This is mostly caused by the fact that both tax rates and dividends only apply when there is profit. Therefore, the positive influence of an increase in ethanol prices on the IRR is attenuated by both of them and the second derivative of these curves is negative.

**Figure 7:**
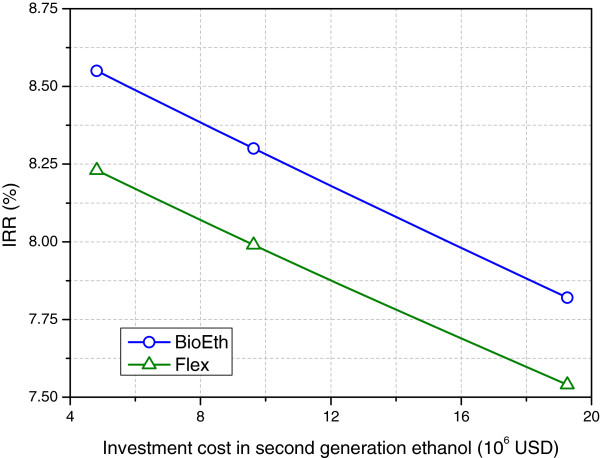
**Impact of the investment cost on second generation ethanol on the internal rate of return.** All other prices kept unchanged.

Since there are many uncertainties regarding the investment costs on the second generation ethanol production process, a sensitivity analysis was also performed for this parameter. Maximum and minimum values of twice and half the base investment cost for the 2G ethanol process were considered. As seen in Figure 7, the impact of the investment cost on the IRR is small. The decrease in IRR between the maximum and minimum investment costs was only of -0.72% and -0.68% for BioEth and Flex, respectively. Therefore, it is expected that the uncertainty of this value will not invalidate the analyses performed.

It is worth highlighting that the 2G process is coupled to the 1G process, which is responsible for the low investment cost of the former, when compared to literature [15], because costs of fermentation, distillation and combined heat and power stages are included in our 1G cost, which is scaled proportionally to the combined process flows.

## Conclusions

A flexible sugarcane biorefinery (Flex) was simulated and compared to dedicated first generation + cogeneration (BioEE) and first + second generation ethanol (BioEth) biorefineries. The flexible one presented an inferior economic performance in all cases for 2012 market prices. Nevertheless, if an increase in electric energy prices in the spot market were considered, the flexible biorefinery could be the best option.

In general, all biorefineries were not economically feasible for 2012 selling prices and costs. This conclusion was actually validated in practice by recent governmental actions (April/2013) which aimed to improve the competitiveness of the ethanol industry. Additionally, of all parameters considered in the sensitivity analysis, ethanol prices were the only ones that could make the biorefineries economically viable, within the studied range of values. In fact, an increase in ethanol price of 21.1% would be sufficient to make feasible all biorefineries. Particularly for BioEth, a 11.5% increase in ethanol prices would be enough for viability.

Enzyme prices, on the other hand, were less significant than it could be expected. This is due to the fact that 2G ethanol costs were diluted by 1G ethanol’s, produced in higher volumetric rates. Therefore, the overall ethanol production cost in the integrated plant was not greatly influenced by enzyme prices.

Finally, it is obvious that the quantitative results presented here are dependent on the economic scenario proposed and on the assumed process yields and energy demands, but the presented methodology is general.

## Methods

### Process implemented

The 1G ethanol production process was simulated based on a typical industrial plant. Figure 1 shows the main required operations. First, sugarcane is cleaned with water to remove dirt carried during harvesting. Next, the sugars are extracted by mechanical pressure. The solution containing the extracted sucrose (juice) follows a series of steps in order to remove impurities which could decrease fermentation yields. The solution is concentrated and fermented by *Saccharomyces cerevisiae*, producing an alcoholic solution which is purified in distillation columns, producing hydrous ethanol. Table 9 shows the main parameters used in the simulations. A rigorous description of the main models used in this study can be found in Furlan *et al.*[16].

**Table 9:** Main data for first generation ethanol production

**Input**	**Value**	**Unit**
Sugarcane flow	500	tonne/h
Sugarcane TRS (Total Reducing Sugars)	15.86	% w/w
**Cleaning section**		
Sugar losses	1.5	%
Cleaning efficiency	70	%
Water flow	1	kg/kg of sugarcane
**Sugar extraction section**		
Sugarcane bagasse humidity	50	% w/w
Sugar recovery (first mill)	70	%
Sugar recovery (total)	96	%
Duty	16	kWh/tonne of fiber
Water flow	30	% w/w
**Sugarcane juice treatment section**		
CaO flow	2	kg/tonne of juice
CaO concentration	10	% w/w
Heating final temperature	105	°C
Steam used	53698	kg/h
Water losses in flash	6495.5	kg/h
Polymer	3534	kg/h
Polymer concentration	0.05	% w/w
Sugar losses (decanter)	6.8	%
Sludge humidity	50	% w/w
Clarifier temperature (after decanter)	92	°C
Sugar losses (filter)	5.6	%
Filter cake humidity	70	% w/w
Water flow (filter)	116	%
**Sugarcane juice concentration section**		
Evaporators area	8000	m^2^
Outlet sugar concentration	21.4	% w/w
Steam consumption	190584	kg/h
Steam produced	181657	kg/h
Pressure of steam produced	2.5	bar
**Fermentation section**		
Fermentation yield	89	%
Yeast concentration (wine)	14	% w/w
Wine ethanol concentration	9	°GL
Yeast concentration (after separation)	70	% w/w
Ration of yeast rich stream / sugar solution	33	% w/w
**Ethanol purification section**		
Specific steam consumption (1G)	2.7	kg/L of ethanol
Specific ethanol production (1G)	91.6	L/tonne of sugarcane
Specific vinasse + phlegm production (1G)	10.1	kg/L of ethanol

The cogeneration system was also considered in the simulations. It uses sugarcane bagasse, sugarcane trash, and alternatively, non-hydrolyzed cellulose and lignin, as fuel and produces steam and electric energy to supply process demands using a Rankine cicle. It was considered that 50% of sugarcane trash is brought from the field to be burnt in the boiler. Since around 140 kg of trash (dry basis) is produced per tonne of sugarcane [9,17], it was considered that a flow of 35 tonnes of sugarcane trash/h was fed to the boiler. If there is a surplus of electric energy, it can be sold to the grid. The cogeneration system includes a boiler, a back-pressure turbine and a condensing one. Table 10 presents the main data used in the simulation of the cogeneration system.

**Table 10:** Main data for the cogeneration system

**Parameter**	**Value**	**Unit**
Cellulose LHV^a^	15997.1	kJ/kg
Hemicellulose LHV^a^	16443.3	kJ/kg
Lignin LHV ^*a*^	24170	kJ/kg
Boiler outlet vapor pressure	65.7	bar
Boiler outlet vapor temperature	520	°C
Boiler efficiency	92	%
Back-pressure turbine outlet pressure	2.5	bar
Back-pressure turbine efficiency	68	%
Condensing turbine efficiency	70	%

^a^Lower Heating Value (LHV) calculated using data from Wooley and Putsche [18].

2G ethanol was produced via the biochemical route, using weak acid pretreatment and enzymatic hydrolysis. The main steps can be seen in Figure 2. First, bagasse is pretreated with a solution of H_2_SO_4_ (3 wt% at 120°C and 2 bar of pressure). At this point, most hemicellulose is hydrolyzed, increasing cellulose accessibility. A filter (Filter_1) is used to separate the solid fraction from the liquid. The solid fraction is pre-hydrolyzed in a horizontal reactor, in order to decrease mixing power demands and water usage. The second hydrolysis is carried out in a stirred reactor without any further addition of water or enzymes. The solid fraction (non-hydrolyzed cellulose + lignin) is separated from the glucose solution in a filter and sent to the boiler to increase steam production. On the other hand, the liquid fraction is directed to the concentration step, being mixed to the 1G juice. The liquid fraction from Filter_1 is sent to a (SIF) reactor, where the xylose in the solution is transformed to xylulose and fermented by *Saccharomices cerevisiae*[19]. The resulting alcoholic solution is sent to the distillation columns with the wine from hexose fermentation. The parameters used for this section are shown in Table 11.

**Table 11:** Main data for second generation ethanol production

**Main data used in the simulation**	**Value**	**Unit**
**Pretreatment**		
Pressure	2	Bar
Temperature	121	°C
Cellulose to glucose conversion	8.0	%
Hemicellulose to xylose conversion	74.0	%
Solid/liquid ratio	0.2	
Acid solution concentration	3	wt%
Volumetric power (mixing)^a^	342	W/m^3^
Space-time	40	min
Reactor volume	182	m^3^
**Pre-hydrolysis**		
Cellulose to glucose yield	20	%
Solid/liquid ratio	0.2	
Enzyme/Cellulose ratio	67.34 (20)	g/kg (FPU/g)
Space-time	18	h
Temperature	50	°C
**Hydrolysis**		
Cellulose to glucose yield	65	%, w/w
Solid/liquid ratio	0.178	
Volumetric power (mixing) ^*a*^	302.5	W/m^3^
Enzyme/Cellulose ratio	67.34 (20)	g/kg (FPU/g)
Space-time	54	h
Temperature	50	°C
**C5 Fermentation**		
Xylose to ethanol yield	70	%, w/w
Temperature	30	°C
Space-time	9	h

^a^Calculated using data from Pereira et al. [20].

### EMSO Software

EMSO [13] was the software chosen as the platform for the simulations in this study. It is an equation-oriented, general purpose process simulator with its own modelling language [21]. Besides the several models for the main process pieces of equipments, the software also allows the user to implement his/hers own models. The software has several numeric solvers for solution of algebraic and differential-algebraic systems, and users can plug in their own numerical routines (in C/C++ or FORTRAN). Physical and thermodynamic properties can be added to the package database by the user whenever needed.

### Economic analysis

Table 12 shows the main economic premisses. Except for 2G ethanol costs, all process costs were obtained from industry (at Dez/2012). 2G ethanol cost is composed by enzyme prices plus all other costs, which are assumed equivalent to 1G’s, due to the lack of industrial information on this topic. Ethanol and electric energy (spot market) selling prices were considered as the mean value over the period between Jan/2003 and Dez/2012. All values were adjusted for inflation in Brazil in the period and converted to US dollars using the exchange rate of 2.077 BRL/USD (dez/2012). For the flexible biorefinery, the economic analysis was made in a monthly basis and, for this case, both ethanol and electric energy price variations due to seasonality were considered (Table 6).

**Table 12:** Economic data, base case used as reference

**Process and economic data**	**Value**
Time usage	80%
Days of operation	210 days/year
Ethanol direct/indirect costs (1G)	94.75 USD/m^3^
Sugarcane costs (1G)	314.78 USD/m^3^
Ethanol production cost(2G, extra cost)	290.1 USD/m^3^
Electric energy production cost	38.9 USD/MWh
Ethanol transportation cost	28.9 USD/L
Administrative and general costs	1.1 USD/TC
Ethanol selling price	513.7 USD/m^3^
Electric energy selling price (public auction)	69.2 USD/MWh
Electric energy selling price (spot market)	40.1 USD/MWh
Enzymes	1.68 USD/kg
Depreciation	10%(p.y.)
Minimum acceptable rate of return	11%(p.y.)
Decrease in production cost due to learning curve*	0.3(1)%(p.y.)
Tax rate (income and social contributions)	34%

^*^for 1G(2G).

## Abbreviations

1G: First generation; 2G: Second generation; BioEE: First generation biorefinery using all sugarcane bagasse (and trash) to produce electricity in a Rankine cicle; BioEth: First generation biorefinery using sugarcane bagasse surplus to produce second generation ethanol; EE: Electric energy; EMSO: Environment for Modeling Simulation and Optimization; Flex: Flexible biorefinery capable of operating as both BioEE and BioEth; IRR: Internal Rate of Return; MARR: Minimum Acceptable Rate of Return; NPV: Net Present Value; SIF: Simultaneous Isomerization and Fermentation; TB: Tonne of bagasse; TC: Tonne of sugarcane.

## Competing interests

The authors declare that they have no competing interests.

## Authors’ contributions

FF did the process simulations in EMSO, the 2G sector economic analysis and the sensitivity tests. RT provided the economic analysis tools and did the economic analysis in the base case, for the 1G sector. FP provided the basic configuration of the 1G plant and helped with the economic analysis. RLG helped defining the technological options for the 2G process configuration (pretreatment, hexoses and pentose fermentation). CC, AC and RG worked in process integration (1G + 2G + EE). RG coordinated the work and supervised all other tasks. All authors read and approved the final manuscript.

## References

[B1] Macedo IC Seabra JEA Silva JEAR Green house gases emissions in the production and use of ethanol from sugarcane in Brazil: The 2005/2006 averages and a prediction for 2020 Biomass Bioenergy 2008 32 582 595 10.1016/j.biombioe.2007.12.006

[B2] Zanin GM Santana CC Bon EPS Giordano RLC Moraes FF Andrietta SR Neto CCC Macedo IC Fo DL Ramos LP Fontana J Brazilian bioethanol program Appl Biochem Biotechnol 2000 84 1147 1163 10849865 10.1385/abab:84-86:1-9:1147

[B3] National Association of Motor Vehicles (ANFAVEA) Brazilian automotive industry yearbook Tech. rep., São Paulo, 2012

[B4] Gnansounou E Production and use of lignocellulosic bioethanol in Europe: Current situation and perspectives Bioresour Technol 2010 101 4842 4850 10.1016/j.biortech.2010.02.002 20194021

[B5] Hassuani SJ Leal MRLV Macedo IC Biomass power generation: Sugarcane bagasse and trash 2005 Piracicaba: United Nations Development Programme and Sugarcane Technology Centre

[B6] Electric Energy National Agency – ANEEL (in portuguese) http://www.aneel.gov.br

[B7] Electric Energy Commercialization Chamber – CCEE (acronym in portuguese) http://www.ccee.org.br/

[B8] Center of Advanced Studies in Applied Economy – CEPEA/ESALQ/USP (in portuguese) http://www.cepea.esalq.usp.br

[B9] Seabra JE Macedo IC Comparative analysis for power generation and ethanol production from sugarcane residual biomass in Brazil Energy Policy 2011 39 421 428 http://www.sciencedirect.com/science/article/pii/S0301421510007706 10.1016/j.enpol.2010.10.019

[B10] Dias MO Cunha MP Jesus CD Rocha GJ Pradella JGC Rossell CE Maciel Filho R Bonomi A Second generation ethanol in Brazil: Can it compete with electricity production? Bioresour Technol 2011 102 19 8964 8971 10.1016/j.biortech.2011.06.098 21795041

[B11] Macrelli S Mogensen J Zacchi G Techno-economic evaluation of 2 nd generation bioethanol production from sugar cane bagasse and leaves integrated with the sugar-based ethanol process Biotechnol Biofuels 2012 5 22 10.1186/1754-6834-5-22 22502801 PMC3350453

[B12] Dias M Junqueira T Cavalett O Cunha MP Jesus C Rossell C Filho R Bonomi A Integrated versus stand-alone second generation ethanol production from sugarcane bagasse and trash Bioresour Technol 2012 103 152 161 10.1016/j.biortech.2011.09.120 22019267

[B13] Soares RP Secchi AR EMSO: A new environment for modelling, simulation and optimisation Comput Aided Chem Eng 2003 14 947 952

[B14] Agblevor F Rejai B Wang D Wiselogel A Chum H blueInfluence of storage conditions on the production of hydrocarbons from herbaceous biomass Biomass Bioenergy 1994 7 213 222 10.1016/0961-9534(94)00063-Y

[B15] Eggeman T Elander RT Process and economic analysis of pretreatment technologies Bioresource Technol 2005 96 18 2019 2025 10.1016/j.biortech.2005.01.017 16112490

[B16] Furlan FF Costa CBB Fonseca GC Soares RP Secchi AR Cruz AJG Giordano RC Assessing the production of first and second generation bioethanol from sugarcane through the integration of global optimization and process detailed modeling Comput Chem Eng 2012 43 1 9

[B17] Canilha L Chandel AK Suzane dos Santos Milessi T Antunes FAF Luiz da Costa Freitas W das Graças Almeida Felipe M da Silva SS Bioconversion of sugarcane biomass into ethanol: An overview about composition, pretreatment methods, detoxification of hydrolysates, enzymatic saccharification, and ethanol fermentation J Biomed Biotechnol 2012 2012 1 15 23251086 10.1155/2012/989572 PMC3516358

[B18] Wooley R Putsche V Development of an ASPEN PLUS physical property database for biofuels components NREL, Tech. rep., Report MP-425-20685 1996, 38

[B19] Silva C Zangirolami T Rodrigues J Matugi K Giordano R Giordano R An innovative biocatalyst for production of ethanol from xylose in a continuous bioreactor Enzyme Microb Technol 2012 50 35 42 10.1016/j.enzmictec.2011.09.005 22133438

[B20] Pereira LTC Pereira LTC Teixeira RSS Bon EPS Freitas SP Sugarcane bagasse enzymatic hydrolysis: rheological data as criteria for impeller selection J Ind Microbiol Biotechnol 2011 38 8 901 907 10.1007/s10295-010-0857-8 20844924

[B21] Rodrigues R Soares RP Secchi AR Teaching chemical reaction engineering using EMSO simulator Comput Appl Eng Educ 2010 18 4 607 618 10.1002/cae.20255

